# Lung Cancer Risk and Past Exposure to Emissions from a Large Steel Plant

**DOI:** 10.1155/2013/684035

**Published:** 2013-11-13

**Authors:** Oscar Breugelmans, Caroline Ameling, Marten Marra, Paul Fischer, Jan van de Kassteele, Johannes Lijzen, Arie Oosterlee, Rinske Keuken, Otto Visser, Danny Houthuijs, Carla van Wiechen

**Affiliations:** ^1^National Institute for Public Health and the Environment, P.O. Box 1, 3720 BA Bilthoven, The Netherlands; ^2^Community Health Service Kennemerland, P.O. Box 5514, 2000 GM Haarlem, The Netherlands; ^3^Comprehensive Cancer Centre Netherlands, P.O. Box 9236, 1006 AE Amsterdam, The Netherlands

## Abstract

We studied the spatial distribution of cancer incidence rates around a large steel plant and its association with historical exposure. The study population was close to 600,000. The incidence data was collected for 1995–2006. From historical emission data the air pollution concentrations for polycyclic aromatic hydrocarbons (PAH) and metals were modelled. Data were analyzed using Bayesian hierarchical Poisson regression models. The standardized incidence ratio (SIR) for lung cancer was up to 40% higher than average in postcodes located in two municipalities adjacent to the industrial area. Increased incidence rates could partly be explained by differences in socioeconomic status (SES). In the highest exposure category (approximately 45,000 inhabitants) a statistically significant increased relative risk (RR) of 1.21 (1.01–1.43) was found after adjustment for SES. The elevated RRs were similar for men and women. Additional analyses in a subsample of the population with personal smoking data from a recent survey suggested that the observed association between lung cancer and plant emission, after adjustment for SES, could still be caused by residual confounding. Therefore, we cannot indisputably conclude that past emissions from the steel plant have contributed to the increased risk of lung cancer.

## 1. Background

Residents living in the surrounding area of a large steel plant located near the Dutch town of IJmuiden have been concerned about health effects from plant emissions for some time. This led to studies into the acute effects of air pollution in the vicinity of the plant [1, 2]. But concerns were augmented by a television documentary broadcast in May 2008, which presented results from a study of metal concentrations in hair from children living in the area [3]. Earlier, in 2007, the Community Health Service reported an increased lung cancer incidence for the region as a whole and for the nearby municipality of Beverwijk in particular [4].

In a large number of studies the health of the residents in the vicinity of steel plants has been investigated. The health effects of long-term exposure include, among, others lung cancer and mortality [5–21]. We present the results from a study, which aims to investigate the spatial distribution of lung cancer incidence rates in a large area around the industrial site at a lower aggregation level than was previously available for studies into the health effects of the steel plant [4]. The study furthermore addresses the influence of smoking habits on lung cancer incidence and aims to assess the association between air pollution from the steel mill and observed lung cancer incidence rates.

## 2. Method

### 2.1. Study Area and Population

**Figure 1:**
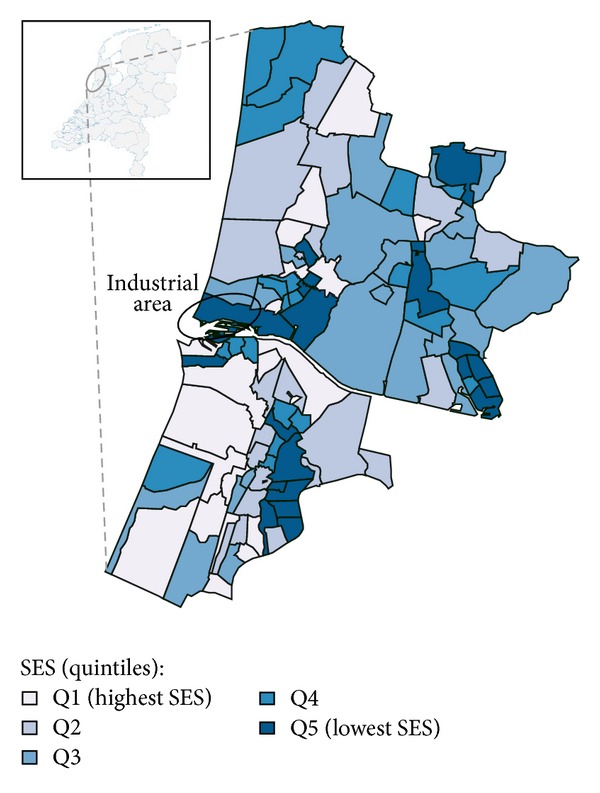
Study area and socioeconomic status. Location of the industrial area within the study area and socioeconomic status (SES—quintiles) of the postcode areas.

The steel plant is located at the west coast of The Netherlands in the IJmond region, covering a terrain of 750 hectares. The plant was developed from 1920 onwards, first producing iron, later steel, and in addition aluminium. The plant consists of 16 factories, including blast furnaces and coke ovens, and has its own harbour. The selected study area surrounds the plant location to the south, east, and north (Figure 1). The study area was selected based on the service area of the Community Health Service that originally investigated the public concern. It is an area in which south-westerly winds prevail, which makes it plausible that plant emissions tend to distribute in a north-eastern direction. The study area was expanded with the inclusion of additional postcodes in this direction. The final study area consists of 17 municipalities and comprises 106 4-digit postcode areas with a total population just below 600,000. Population data at the postcode level were available from Statistics Netherlands (CBS) stratified by age and sex.

### 2.2. Air Pollution Data

Plant emissions have been recorded since 1985. Historic emission data for the period 1950–1984 were obtained by combining the emission registration data from 1985 with historic production data. Polycyclic aromatic hydrocarbons (PAH), lead, and cadmium were the relevant and available air pollution indicators for the health effect under study. The concentrations of these components were modelled for seven (PAH) or eight (metals) consecutive periods from 1950 to 2007 for a 15 × 15 km model area at a 100 × 100 m resolution [22]. The period lengths varied from 3 to 23 years according to assumed invariance of emissions. From the geographical distributions of the concentrations, we calculated population density weighted average concentrations for each postcode situated (for the greater part) within the air pollution modelling area. Subsequently, the postcode areas were divided into four exposure categories based on quartiles.  Figure 3 shows that the air pollution modelling area covers only part of the total study area; the remaining 63 postcodes outside the modelling area constituted the fifth (reference) category. Effects of air pollution concentrations due to steel plant emissions were assumed to be negligible for these areas.

### 2.3. Lung Cancer Data

Yearly lung cancer incidences (ICD10 C34) were obtained for a consecutive period of 12 years (1995–2006) from the Comprehensive Cancer Centre Amsterdam (IKNL). Since 1989, IKNL maintains the only Dutch oncology registry with a coverage of at least 95% of all cancer patients. The study period was started in 1995 as age and gender specific population data at postcode level have become available since then. Due to privacy regulations, the observed number of lung cancer cases cannot be reported below the postcode aggregation level, making this the aggregation level of choice for the analysis.

### 2.4. Data on Potential Confounders

Cancer risk is determined to a large extent by individual lifestyle factors such as smoking, diet, alcohol use, and obesity. For lung cancer risk, smoking behaviour is the dominant factor that might influence the results of this study. As no historical data on lifestyle factors were available at postcode level for the study area as a whole, we used socioeconomic status (SES) as a proxy to adjust for potential confounding. SES categories constructed from level of income, education, and professional status are available for every 4 years since 1994 from The Netherlands Institute for Social Research [23]. We used percentile values for postcode areas based on the ranking order for SES of all postcode areas in The Netherlands. In addition, we obtained data from recent health surveys among 6,044 adults (19–65 years) and 5,691 elderly (over 65 years) for 2007 and 2008 on current and past smoking habits that was conducted for part of the study area (67 postcode areas) by one of the Community Health Services [24, 25]. With this survey data, the prevalence of current and past smoking was calculated per postcode area. Comparison of smoking prevalence and SES in a smaller part of the study area served as a check on the ability of the SES indicator to serve as a proxy for the differences in smoking behaviour between postcodes.

### 2.5. Data Analysis

The expected number of lung cancer cases per postcode area was calculated through indirect standardization, based on the age and gender distribution of the population in a postcode and using the age and gender distribution of the lung cancer cases for the whole study region as the reference population. As second step, a Poisson regression model with indicator variables for each year was applied. This model was subsequently extended to a Bayesian hierarchical Poisson regression model with a conditional autoregressive spatial correlation structure to determine spatially smoothed expected incidences [26–28]. As a fourth step, the regression model was adjusted for SES. Maps of spatially smoothed standardized incidence ratios of observed and expected rates (SIR) for the postcode areas were produced to assess the spatial pattern of lung cancer incidence for the population as a whole as well as for men and women separately. To assess the association between air pollution indicators from the steel mill and observed lung cancer incidence rates, the 5 exposure categories for PAH and cadmium were added to separate regression models. We evaluated the effect of exposure on lung cancer incidence as relative risk (RR). To evaluate potential residual confounding by smoking after adjustment for SES, we determined whether a relation between exposure and smoking was present in part of the study area and whether this relationship still existed after adjusting for SES.

## 3. Results

The average yearly population for the area consisted of 290,160 men and 303,860 women, while the average population within the postcodes amounted to 5,600 ranging from approximately 100 inhabitants up to almost 16,000 inhabitants. The total number of lung cancer cases during the 12-year period was 3,029 for men and 1,388 for women (4,417 cases in total). The yearly lung cancer incidence for the population in the study area was on average 5% lower than for the total population in The Netherlands.

**Figure 2:**
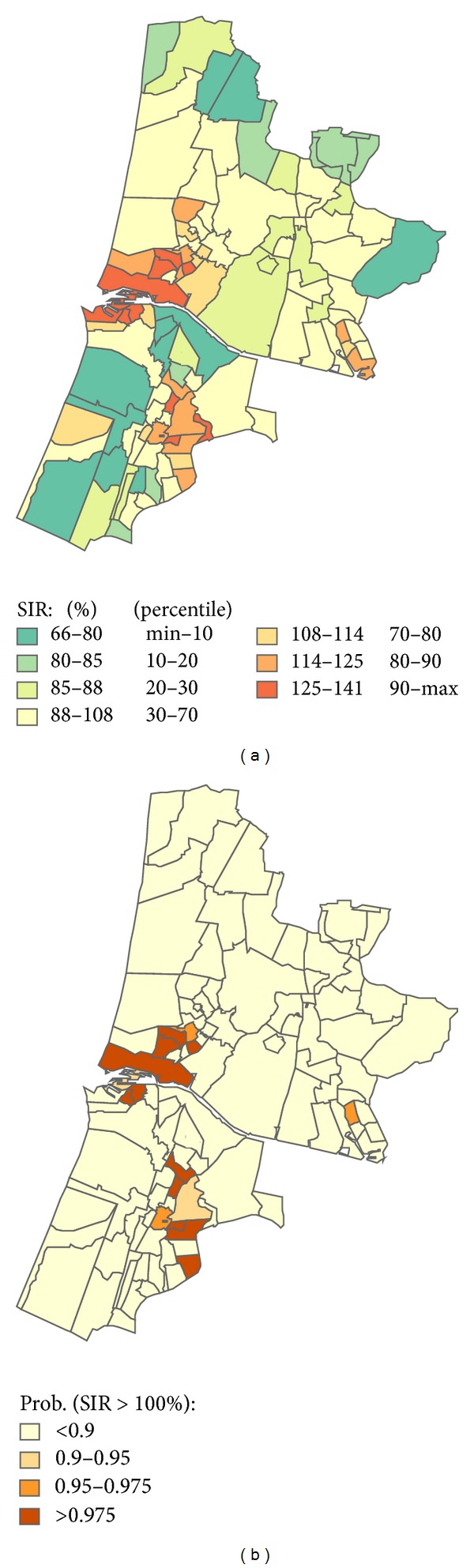
Lung cancer incidence. Standardized incidence ratios (SIR) for lung cancer within each postcode area (left) and the statistical probability that the SIR of a postcode area is higher than expected.

The distribution of the standardized incidence ratios (SIR) and the effect of spatial smoothing are shown in Table 1. After spatial smoothing, lung cancer showed significant increases in the SIR of up to 40% in postcode areas in 2 municipalities located within 5 km from the industrial area. Similar increases were seen in parts of an urban area in the southern part of the study area further away from the industrial complex (Figure 2). 

**Figure 3:**
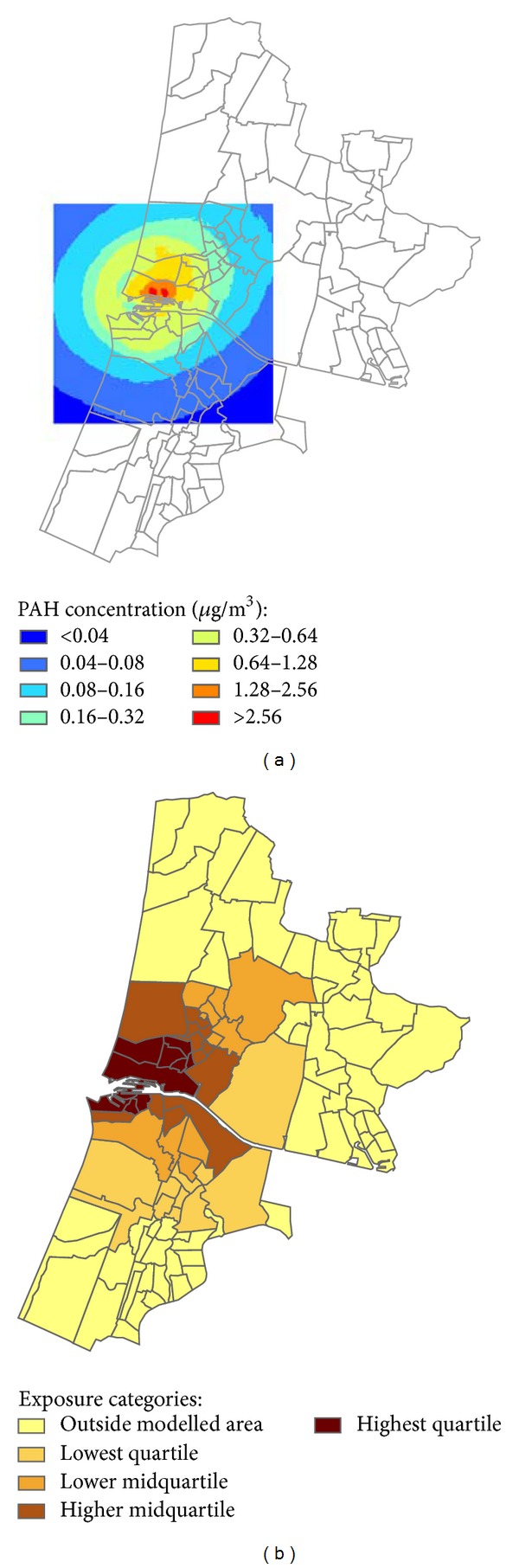
Average PAH concentrations. Modelled concentrations of polycyclic aromatic hydrocarbon (1972–1994) and allocation of postcode area to exposure categories based on the average PAH concentration within the postcode areas.

There was very little or no discernible difference in ranking of postcode areas over time for both PAH and metal concentrations. Similarly, there was little or no discernible difference in ranking of postcode areas between lead and cadmium concentrations. Therefore, analyses were performed using average data from a single time period (1972–1994) for PAH and cadmium only. The exposure categories for PAH and cadmium did not exactly coincide due to different locations of the emission sources of PAH and cadmium at the plant site. On the left side, Figure 3 shows the geographical distribution of the modeled average PAH concentrations for the period 1972–1994 and the location of the modelling area within the total study area. The right side of Figure 3 shows how the postcodes that are located completely or partly within the modelling window were assigned to the four exposure categories based on the average PAH concentrations of the postcodes. The remaining 63 postcodes outside the modelling window were assigned to the reference category.

The distribution of the SES categories (quintiles) across the study area is shown in Figure 1. On average, the SES scores in the study area were 8% lower than within The Netherlands as a whole. However, the SES scores covered almost the full scale ranging from the 5th to the 99th percentile. SES scores for a single point in time could be used in the analysis because SES ranking of the postcodes hardly varied over the years during the study period (1995–2006). The relative risks (RR) for lung cancer in postcodes within the lowest SES quintile compared to postcodes within the highest SES quintile were 1.59 (95%CI: 1.36–1.85) for men and 1.83 (95%CI: 1.44–2.29) for women. Over the twelve-year study period (1995–2006), SES ranking for postcode areas varied little over the years.

**Figure 4:**
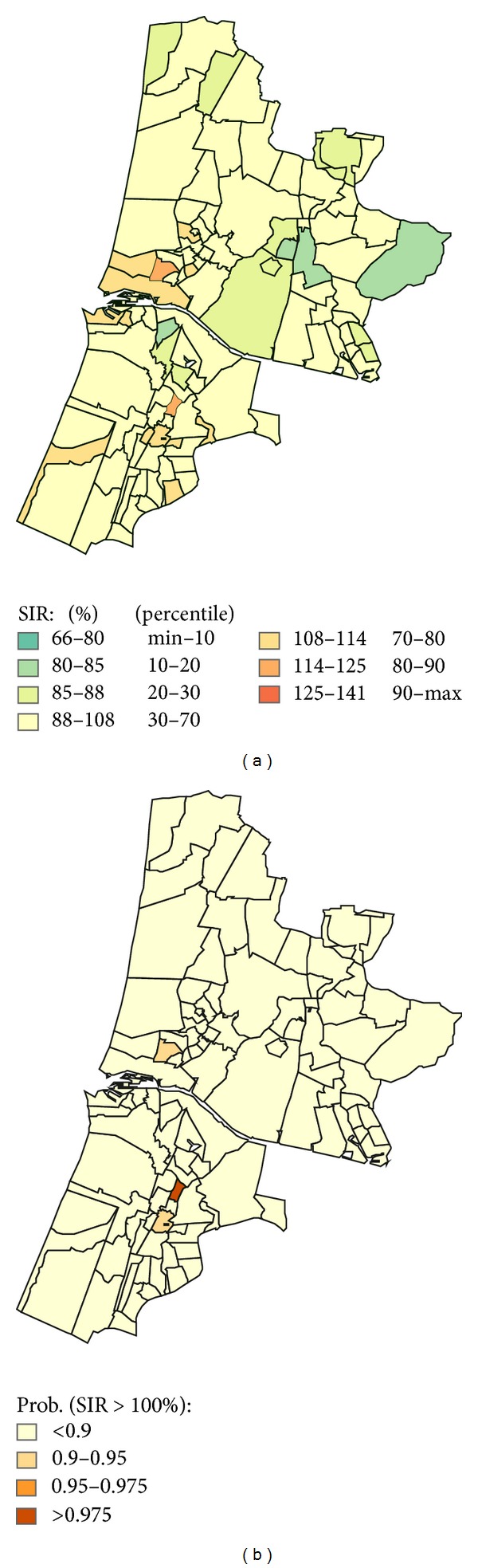
Lung cancer incidence—correction for socioeconomic status. SIR for lung cancer within each postcode area after correction for the influence of differences in SES (left) and the statistical probability that the SIR of a postcode area is higher than expected.

The PAH exposure range, average population per year during the study period, and the total number of lung cancer cases are given in Table 2 for each exposure category. The table also shows the RR for lung cancer in each exposure category and the effect of smoothing and correction for SES on the RR. The RR to develop lung cancer for those living in postcode areas in the highest PAH exposure category compared to those living outside the exposure modelling area is 1.35 (95%CI: 1.23–1.48). The effect size diminishes slightly after applying spatial smoothing and adjustment for SES resulting in a RR of 1.21 (95%CI: 1.01–1.40) for men and women combined. The RR in the highest exposure category is 1.22 (95%CI: 1.02–1.50) for men and 1.16 (95%CI: 0.87–1.50) for women in the model taking into account spatial smoothing and adjustment for SES. The RR was significantly increased for men living in the highest exposure category only. The SIR for lung cancer at postcode level after correction for SES is presented in Figure 4. Compared to Figure 2, the number of postcodes with a SIR that is higher than expected is reduced.

Similar results were found for cadmium (Table 3). The RR in the highest exposure category decreased from 1.34 (95%CI: 1.21–1.48) to 1.23 (95%CI: 1.03–1.50) after adjustment for SES and applying spatial smoothing for men and women combined. Again the results for men and women separately were comparable: 1.22 (95%CI: 1.01–1.50) for men only and 1.26 (95%CI: 0.94–1.70) for women only. Statistically significant elevated RRs due to exposure to air pollution from the industrial area were only found in the highest exposure category.

As SES can only serve as a proxy for smoking behaviour, potential residual confounding was further analyzed using an external data source on smoking habits, which covers only part of the study area and which was collected by one of the Community Health Services after the time window of our study. In the highest air pollution exposure category, among women younger than 65 years of age, the odds ratio (OR) for “current smoking” after adjusting for SES is 1.34 (95%CI: 1.09–1.64), indicating that the prevalence of female smokers in this air pollution exposure category is higher than in the reference area and even higher than could be expected on the basis of lower SES. In addition, in the highest air pollution exposure category among men of 65+ years of age an increased OR after adjusting for SES of 1.82 (95%CI: 1.20–2.75) was observed for “ever smoking.” 

## 4. Discussion 

In this study, we found that the SIR for lung cancer was up to 40% higher than average in postcode areas in two municipalities in the proximity of the industrial terrain of a steel plant. With a total number of over 4,000 lung cancer cases over a period of twelve years, this study was relatively large in comparison to similar research from other scientific publications. We found two studies of similar size [11, 17]. The first concerned the mortality risk of residents living near 22 coke plants in England, Scotland, and Wales over the period 1981–1992 and reported a small but significant excess mortality risk of 3% for residents living within 2 kilometres of the coke plants, after correction for SES. This is considerably less than the 21% excess risk found in this study. The second describes lung cancer risks due to a coke oven plant near Genoa, Northern Italy. Only a marginal excess risk was found in the exposed area compared to the two reference areas. Published ecological studies with a smaller number of cases tend to report higher relative risks than we found, albeit with much larger confidence intervals. Extensive research has been carried out around the steel foundries in Armadale, Bathgate, and Kirkintilloch in Scotland using both ecological and case-control study designs [15, 16, 20, 21, 29]. The researchers attributed the increased cancer mortality risk that was found to a change in production process of the plants in the sixties. Due to the small number of cancer cases this was difficult to confirm using the available statistical methods at the time. Later research [14] did indeed find a lung cancer cluster using more advanced statistical methods. Ecological research around the large complex of steel and petrochemical plants near Teesside indicated increased lung cancer mortality over the period 1981–1991 in areas with increased exposure to air pollution [9, 18]. This was confirmed by a subsequent case-control study that found, after correction for confounding factors, a RR of 1.83 (95%CI: 0.82–4.08) for women living more than 25 years near heavy industry and 1.10 (95%CI: 0.96–1.26) for women living there more than 10 years. Similar ecological research near a coke plant in South Tyneside over the period 1981–1989 did not show elevated lung cancer risks [8]. The finding that length of residence influences the cancer risk is an important one. However, this factor is difficult to take into account using an ecological study design. It was not possible to assess and account for the migration patterns of the population in our study. 

Results from studies in North America show inconsistent findings. Archer studied lung cancer mortality risks in 3 communities in Utah and estimated an increase of 30–40% due to air pollution from a steel factory over the period 1950–1987 [5]. However, a similar study carried out in the same area did not find increased mortality risks after standardizing for smoking behaviour [10]. Both studies have been found sensitive for the way confounding factors were taken into account and the construction of control areas [19]. Several studies have been carried out in Sydney (Nova Scotia, Canada) around a coke plant and steel foundry [6, 7, 13]. Over a period of 45 years, mortality in Sydney was higher for breast and intestinal cancer compared to Canadian reference figures. In three highly exposed areas near the plants, an increased lung cancer risk was found with a standard mortality ratio of 1.41 for men (95%CI: 1.11–1.77) and 1.76 for women (95%CI: 1.13–2.63). A subsequent case-control study could not confirm these findings due to the small number of cases that could be reached.

Older studies do not correct for SES. Those studies that do correct for SES or smoking habits like in our study find similar decreases in relative risks. A few case-control studies have been published [6, 12, 16]. The latter was the only study that produced information on relative risks for lung cancer in relation to living near an industrial area including a steel plant. For 204 female lung cancer cases and 339 controls they found a RR for lung cancer of 1.83 (95%CI: 0.82–4.08) at 25 years of residence and of 1.10 (95%CI: 0.96–1.26) at 10 years of residence.

The PAH and cadmium concentrations being modelled on the basis of emission data introduce uncertainty in the estimated absolute levels of historical environmental concentrations. As we were interested in the contrast in health effects between high and low exposed areas, the relative ranking of the postcode areas within the study area is of more importance than the absolute levels of the pollutant concentrations. This still leaves the possibility of exposure misclassification of postcodes due to uncertainties in the emission data and the modelling exercise, especially at larger distances from the industrial site where meteorological and model assumptions play a larger role on the outcome of the emission model. Since the observed effects only occur in the highest exposure category, the validity of the exposure contrast between the reference and the highest exposure category is most critical. As we expect the least misclassification in the highest exposure category, the misclassification in the reference category is the most relevant. However, as the reference category is very large consisting of 62% of the study population and 60% of the cancer cases, it is unlikely that misclassification of postcodes in this category will affect the baseline incidence rate in the reference category and thereby the RR.

The available data for the pollutants show a high degree of correlation in concentration across time and space [30]. Because of this correlation, it is not possible to distinguish between individual effects of each pollutant. The data for PAH and cadmium used must therefore be regarded as representative for historical exposure to the total air pollution from the steel plant. We did not investigate alternative sources of air pollution, such as other nearby industries or shipping. In a sensitivity analysis (not presented here), we investigated levels of air pollution from NO_2_ and PM10, which can be attributed to various local sources [31–33]. We found no relation between these air pollution levels and the increased lung cancer incidences. As these concentrations of NO_2_ and PM10 date from the start of this century, we cannot preclude that earlier air pollution from local sources may have contributed to the increased lung cancer risks found. If historical pollution from other sources was correlated with the PAH and cadmium concentrations from the steel plant which we used, it is possible they also contributed to the resulting increased risk. 

At the postcode level, SES was the only available confounder that corresponded with the studied time window of the incidence of lung cancer. We are aware that the use of SES does not warrant a full adjustment for smoking habits. It is also possible that changes in smoking habits over time did not correspond to the SES strata in the studied time window [34]. Where this leads to misclassification relating in any way to exposure, residual confounding may occur. That residual confounding indeed may be present is suggested by results from an analysis of the only available additional smoking data, which covers only a part of the study area and which was collected after the time window of our study. The analysis shows that in some age categories higher smoking percentages were found in the highest exposed postcode areas than were expected based on SES for these areas.

Occupational exposure was not addressed in this study. As there is little difference between the relative risks found for both sexes, the increased lung cancer incidences are less likely to be explained by occupational exposure.

The ecological epidemiological design of the study allowed us to analyse the geographical distribution of lung cancer incidence for a large population in relation to certain risk factors. The lack of information at the individual level means that conclusions can only be interpreted at the group level. The increase in relative risk is an indication that possible past exposure to air pollution from the steel plant may have led to an increase in lung cancer incidence over the period 1995–2006. It is not certain whether SES provides a sufficient adjustment for past smoking habits. In addition, there is lack of information on past levels of PM10 and other components from other sources of air pollution. Contribution of these other factors to the increase in relative risk can therefore not be excluded. 

## 5. Conclusion

We observed an increased lung cancer incidence in certain postcode areas near the steel plant, after adjustment for SES. In the areas in which the highest historical exposure to PAH and cadmium occurred, lung cancer incidence after adjustment for SES was increased by 21% over the average incidence for the study area. We were unable to ascertain that adjustment for SES fully compensates for the effect of smoking. Due to the possible residual confounding of smoking and the limited availability of and uncertainties in the historical exposure data, we cannot indisputably conclude that past emissions from the steel plant have contributed to an increased risk of lung cancer.

## Figures and Tables

**Table 1:** Standardized incidence ratios for lung cancer.

SIR	Age standardization	Age + spatial smoothing	Age + spatial smoothing + SES
Mean	1.01	1.00	0.97
Median	0.97	0.97	0.97
Standard deviation	0.43	0.17	0.08
Minimum	0.00	0.66	0.81
Maximum	3.55	1.41	1.20
Interquartile range	0.79–1.21	0.86–1.12	0.90–1.04

Distribution of SIRs for lung cancer within the study area after indirect standardization for age and sex, spatial smoothing, and correcting for the influence of SES.

**Table 2:** Relative risks for lung cancer in relation to PAH exposure.

	Range PAH conc. period 1972–1994 (µg/m3)	Average population per year	No. of cases in 12 years	RR without smoothing [95% CI]	RR after smoothing without SES [95% CI]	RR after smoothing and SES correction [95% CI]
Outside modelled area	n.a.	370,259	2,646	1	1	1
First quartile	0.032–0.052	72,962	533	1.07 [0.97–1.17]	1.02 [0.85–1.23]	1.05 [0.91–1.20]
Second quartile	0.055–0.159	63,508	380	0.91 [0.82–1.01]	0.91 [0.73–1.12]	0.93 [0.80–1.10]
Third quartile	0169–0.390	45,911	364	1.09 [0.98–1.22]	0.98 [0.76–1.25]	1.03 [0.86–1.20]
Fourth quartile	0.426–0.636	46,931	494	1.35 [1.23–1.48]	1.27 [0.97–1.66]	1.21 [1.01–1.40]

RR for lung cancer in relation to PAH exposure from the lowest (first) to the highest (fourth) exposure quartiles with and without smoothing and correction for SES.

**Table 3:** Relative risks for lung cancer in relation to cadmium exposure.

	Range cadmium conc. period 1973–1984 (ng/m3)	Average population per year	No. of cases in 12 years	RR without smoothing [95% CI]	RR after smoothing without SES [95% CI]	RR after smoothing and SES correction [95% CI]
Outside modelled area	n.a.	370,259	2,646	1	1	1
First quartile	0.30–0.42	60,837	443	0.97 [0.88–1.07]	0.90 [0.74–1.09]	0.99 [0.85–1.10]
Second quartile	0.42–0.72	69,253	381	0.93 [0.84–1.04]	0.95 [0.79–1.15]	0.95 [0.83–1.10]
Third quartile	0.73–0.87	57,464	501	1.19 [1.08–1.31]	1.26 [1.01–1.61]	1.11 [0.94–1.30]
Fourth quartile	0.89–1.65	41,758	446	1.34 [1.21–1.48]	1.38 [1.06–1.83]	1.23 [1.03–1.50]

RR for lung cancer in relation to cadmium exposure from the lowest (first) to the highest (fourth) exposure quartile with and without smoothing and correction for SES.
